# CD147: an integral and potential molecule to abrogate hallmarks of cancer

**DOI:** 10.3389/fonc.2023.1238051

**Published:** 2023-11-07

**Authors:** Alphonce M. K. Nyalali, Angela U. Leonard, Yongxiang Xu, Huayu Li, Junlin Zhou, Xinrui Zhang, Tibera K. Rugambwa, Xiaohan Shi, Feng Li

**Affiliations:** ^1^ Department of Neurosurgery, Shandong Cancer Hospital and Institute, Shandong First Medical University and Shandong Academy of Medical Sciences, Jinan, China; ^2^ Department of Neurosurgery, Qilu Hospital of Shandong University, Cheeloo College of Medicine, Shandong University, Jinan, China; ^3^ Department of Surgery, Songwe Regional Referral Hospital, Mbeya, Tanzania; ^4^ Department of Orthopedics and Neurosurgery, Mbeya Zonal Referral Hospital and Mbeya College of Health and Allied Sciences, University of Dar Es Salaam, Mbeya, Tanzania; ^5^ Department of Pediatrics and Child Health, Mbeya Zonal Referral Hospital and Mbeya College of Health and Allied Sciences, University of Dar Es Salaam, Mbeya, Tanzania; ^6^ Department of Public Health, Muhimbili University of Health and Allied Sciences, Dar es Salaam, Tanzania; ^7^ School of Nursing and Rehabilitation, Shandong University, Jinan, China; ^8^ Department of Oncology, Xiangya Hospital, Central South University, Changsha, Hunan, China; ^9^ Department of Internal Medicine, Mbeya Zonal Referral Hospital and Mbeya College of Health and Allied Sciences, University of Dar Es Salaam, Mbeya, Tanzania

**Keywords:** CD147, EMMPRIN, hallmarks of cancer, cancer enabling characteristics, prooncogenes, targeted cancer therapies

## Abstract

CD147 also known as EMMPRIN, basigin, and HAb18G, is a single-chain type I transmembrane protein shown to be overexpressed in aggressive human cancers of CNS, head and neck, breasts, lungs, gastrointestinal, genitourinary, skin, hematological, and musculoskeletal. In these malignancies, the molecule is integral to the diverse but complimentary hallmarks of cancer: it is pivotal in cancerous proliferative signaling, growth propagation, cellular survival, replicative immortality, angiogenesis, metabolic reprogramming, immune evasion, invasion, and metastasis. CD147 also has regulatory functions in cancer-enabling characteristics such as DNA damage response (DDR) and immune evasion. These neoplastic functions of CD147 are executed through numerous and sometimes overlapping molecular pathways: it transduces signals from upstream molecules or ligands such as cyclophilin A (CyPA), CD98, and S100A9; activates a repertoire of downstream molecules and pathways including matrix metalloproteinases (MMPs)-2,3,9, hypoxia-inducible factors (HIF)-1/2α, PI3K/Akt/mTOR/HIF-1α, and ATM/ATR/p53; and also functions as an indispensable chaperone or regulator to monocarboxylate, fatty acid, and amino acid transporters. Interestingly, induced loss of functions to CD147 prevents and reverses the acquired hallmarks of cancer in neoplastic diseases. Silencing of Cd147 also alleviates known resistance to chemoradiotherapy exhibited by malignant tumors like carcinomas of the breast, lung, pancreas, liver, gastric, colon, ovary, cervix, prostate, urinary bladder, glioblastoma, and melanoma. Targeting CD147 antigen in chimeric and induced-chimeric antigen T cell or antibody therapies is also shown to be safer and more effective. Moreover, incorporating anti-CD147 monoclonal antibodies in chemoradiotherapy, oncolytic viral therapy, and oncolytic virus-based-gene therapies increases effectiveness and reduces on and off-target toxicity. This study advocates the expedition and expansion by further exploiting the evidence acquired from the experimental studies that modulate CD147 functions in hallmarks of cancer and cancer-enabling features and strive to translate them into clinical practice to alleviate the emergency and propagation of cancer, as well as the associated clinical and social consequences.

## 1 Introduction

### 1.1 Tumor heterogeneity and hallmarks of cancer

The dynamics of genetic mutations, acquired cellular plasticity, and tumor microenvironment characteristics are known to drive cancer cells to acquire distinctive genomic and phenotypic features. These not only strikingly differentiate the cancer cells from normal cells of the same tissue but also bring about variations of characteristics between cancer types, the same cancer type in different patients (inter-tumoral heterogeneity), and cellular subpopulations within the same tumor (intra-tumoral heterogeneity) (1, 2). Despite the inter-tumoral and intra-tumoral heterogeneity at the molecular level, the acquired genomic and phenotypic ability enables cancer cells to emanate a similar spectrum of complex or intertwined features and characteristics. These features ensure cancer survival and progression through relatively autonomous cancerous machinery, as well as evasion or hijacking of the limitations and regulatory mechanisms of the surrounding normal cells and physiology. About two decades ago Hanahan and Weinberg distilled the acquired and complex characteristics that dictate and propagate neoplastic transformation of the cellular physiology into malignant growth (3). They recategorized them into six distinct features named hallmarks of cancer. These included self-sufficiency in growth signals, insensitivity to anti-growth signals, evading apoptosis, sustained angiogenesis, limitless replicative potential, and tissue invasion and metastasis (3). A decade later, they proposed two additional hallmarks of cancer: metabolic reprogramming and immune evasion (4). This conceptualization has been embraced with enthusiasm by the scientific community, thus becoming an oasis of research concepts and objectives. Conceptual progress to date proposes additional hallmarks that include phenotypic plasticity or disrupted differentiation (dedifferentiation, blocked differentiation, and trans-differentiation), and nonmutation epigenetic modification. Genomic instability and mutation, polymorphic microbiomes, and tumor-promoting inflammation are retained as ‘cancer enabling characteristics’, even though some studies propose to qualify them as hallmarks of cancer (4–6). The above characteristics form the central hub of cancer mechanisms around which almost all arms of cancer research revolve. They form the Achilles’ heel of malignant diseases henceforth viable targets for effective cancer therapies.

Behind the emanation of the hallmarks of cancer and cancer-enabling characteristics is a repertoire of genes and proteins that form the switches and pathways that drive the transcriptomic and proteomic machinery of cancer. Understanding and exploiting the cancer characteristics therefore demands the in-depth exploration and understanding of individual molecules and their roles in the web of cancer machinery. In this study, we review the role of CD147 in the emanation and propagation of the hallmarks of cancer and cancer-enabling characteristics. We found that CD147 has a regulatory or mediatory role in the upstream and downstream, single, or complex molecules and pathways that propagate most of the hallmarks of cancer and cancer-enabling characteristics. Inducing loss of functions to CD147 could prevent or reverse the acquisition of the hallmarks of cancers in the neoplastic cells and tissues.

### 1.2 CD147, a critical molecule in human pathophysiology

Human Cluster of Differentiation 147 (CD147) also known as extracellular matrix metalloproteinase inducer (EMMPRIN), HAb18G, and basigin, is a single-chain type I transmembrane protein, coded by the *BSG* gene located on chromosome 19p13.3. It belongs to the immunoglobulin superfamily (IgSF) of receptors (7–9). The molecule occurs in four isoforms: basigin 1 which is retinal-specific with three immunoglobulin-like domains, isoforms 3 and 4, each with a single immunoglobulin domain, and isoform 2 which is the most abundant, well-studied, and contains two immunoglobulin domains. The latter is composed of 269 amino acids and its mature protein has a molecular weight of 29kDa and an extractable weight between 35 and 65kDa due to variable degrees of N-terminal glycosylation at Arginine residues (9, 10).

CD147 plays a critical and extensive role in both infectious and non-infectious disease development and/or propagation. In infectious pathologies for example, during malarial parasitic infection, CD147 interacts with Plasmodium falciparum reticulocyte-binding protein homolog 5 (PfRH5) and rhoptry-associated protein 2 (RAP2). This is the essential interaction among the four modes of PfRhs-erythrocyte interactions that occurs during the reorientation stage (second stage) of parasite invasion into the erythrocyte (11, 12); again, in the fourth and final stage of plasmodium falciparum-erythrocyte invasion (parasitophorous vacuole formation), CD147interacts with the rhoptry-associated protein 2 (RAP2). RAP2 and other proteins are involved in the completion of merozoite invagination into the erythrocyte (11). In viral infections such as by SARS-Cov-2 or human immunodeficiency virus 1 (HIV-1), one study reported CD147 to be an alternative receptor to angiotensin-converting enzyme 2 (ACE-2) receptor of the SARS-Cov-2 spike proteins (13). Notably, it is this interaction targeted in the antiviral treatment of SARS-Cov-2 with Meplazumab (14). In HIV-1 infection, CD147 interacts with extracellular cyclophilin A (CyPA), a ligand to CD147 whereby CD147-CyPA interaction enhances CyPA-dependent invasion of white cells by the HIV-1 virus up to 6 folds (15).

CD147 is also integral in non-transmissible pathologies such as stroke, heart disease, and Alzheimer’s disease (9), and more interestingly as a molecule that is extensively and integrally involved in the development, progression, metastasis, and prognosis of numerous human cancers (9, 16). For example, CD147 is differentially overexpressed in glioblastoma (17), carcinomas of the head and neck (18), lungs (19), breasts (20), liver (21, 22), stomach (23), pancreas (24), colorectal (25), renal (26), bladder (27), prostate (28), ovary (29), and cervical (30); as well as in melanoma (31), sarcomas (32), and leukemia (33) (**Table 1**). It is therefore plausible to infer that CD147 has an invaluable role to play in neoplastic cellular transformation, cancer development, progression, and adaptation.

**Table 1:** CD147/HAb18G Expression in normal and malignant tissue, and prognostic indication in systemic-representative aggressive human cancers.

System/Region	Pathology	CD147 IHC Expression Rate, n/N (%)		Prognostic Significance	References
		Pathological tissue	Normal Tissue		
CNS	Glioblastoma	190/206 (92.2)	7/36 (19.4) *p<0.05*	Poor KPS	(17)
Head and Neck	Laryngeal Carcinoma	42/48 (87.5)	4/15 (26.7) *p<0.01*	Metastasis, Poor OS	(18)
Breast	Breast carcinoma (TNBC)	61/127 (48.0)	0/30	Metastasis, Poor OS	(20)
Respiratory	Lung cancer (NSCLC)	173/208 (83.2)	0/41	Poor OS	(19)
Gastrointestinal	Hepatocellular Carcinoma	52/82 (63.4)	–	Poor RFS	(21)
	HBV-related Cirrhosis	65/68 (95.6)	8/125 (6.4) *p<0.0001*	High Child-Pugh grade	(22)
	Gastric cancer	133/223 (59.6)	48/223 (21.5) ^*^ *p<0.001*	Poor PFS and OS	(23)
	Pancreatic Cancer	22/97 (22.7)	–	Poor PFS	(24)
	Colorectal Carcinoma	207/328 (63.1)	56/328 (17.1) *p<0.001*	Metastasis, Poor DFS	(25)
Genitourinary	Epithelial Ovarian Cancer	48/60 (80.0)	1/20 (5.0)	Metastasis, Poor Survival	(29)
	Cervical Carcinoma	67/85 (78.8)	6/24 (25.0), *p<0.0001*	Metastasis	(30)
	Renal Cell Carcinoma	268/394 (68.0)	263/362 (72.7) ^*^ *p=0.991*	Tumor grade, Poor OS	(26)
	Urinary Bladder Carcinoma	52/108 (48.2)	0/20	High grade, Poor Prognosis, and OS	(27)
	Prostate Carcinoma	113/240 (47.1)	1/20 (5.0) *P<0.006*	Poor PSA-Failure Free) Poor Metastasis Free survival and OS	(28)
Musculoskeletal	Osteosarcoma	45/55 (81.8)	0/15	High-stage, Poor OS	(32)
Skin	Melanoma	17/28 (60.7)	0/28	Metastasis	(31)
Hematological	Acute Myeloid Leukemia	52/62 (83.9)	2/20 (10.0), *p=0.001*	Poor RFS and OS	(33)

IHC, Immunohistochemical Staining; CNS, Central Nervous System; TNBC, Triple-negative Breast Cancer; NSCLC, Non-small Cell Lung Cancer; HBV, Hepatitis B Virus, KPS, Karnofsky Performance Score; OS, Overall Survival; _mv_p, p-value from CD147 multivariate (independent) analysis; PFS, Progression Free Survival DFS, Disease Free Survival; RFS, Relapse Free Survival. n, Cases/controls with positive CD147 staining; N, Total cases/controls *, The researchers used peritumoral tissue as study controls.

#### 1.2.1 CD147 and emanations of hallmarks of cancer in numerous tumors

CD147 is integral to the diverse but complimentary hallmarks of cancers. The molecule is pivotal in cancerous proliferative signaling, growth propagation, cellular survival, replicative immortality, angiogenesis, metabolic reprogramming, immune evasion, and invasion and metastasis (34–38). The molecule also has regulatory functions in the cancer-enabling characteristics, including DNA damage response (DDR) and immune evasion. A pool of experimental and clinical studies divulged the critical role of CD147 in the exhibition of one or several hallmarks of cancer and cancer-enabling characteristics in numerous cancer types. Examples of such cancers include glioblastoma (39), retinoblastoma (40), head and neck squamous cell carcinoma (34), breast cancer (35, 36), non-small-cell lung cancer (37), hepatocellular carcinoma (38, 41, 42), pancreatic cancer (43–45), gastric carcinoma (46), colon cancer (47), melanoma (47), cervical cancer (48), and prostate cancer (49, 50).

## 2 Role of CD147 in acquisition and propagation of hallmarks of cancer by neoplastic cells

### 2.1 CD147 orchestrates cell cycle, cellular stemness, and proliferation

One of the key pathological properties of cancer cells is their ability to switch from a quiescent state into a vicious replicative mode associated with the evasion of anti-growth signals (51). To commit to the replicative state, the cells exclusively rely on a repertoire of proteins with diverse functions in cell cycle machinery, stem cell acquisition, and immortality (51). Some of these proteins are D-type cyclins (cyclin D) and cyclin-dependent kinases (CDKs), they mediate the G1 to S transition of cells into the cell cycle whereby Cyclin D-CDK complexes induce hyperphosphorylation of the tumor suppressor retinoblastoma protein (RB), a critical regulator of the cell cycle in normal cells. Hyperphosphorylated RB releases transcription factors E2F which in turn activates a plenitude of cell cycle proteins that commit the cells into vicious replication (52, 53). Upstream to the cyclin and Cdks is CD147, CD147 has been shown to induce phosphorylation of ERK or PI3K-Akt pathways which results in the activation of transcription of the cell cycle driver-proteins. In a study by Obchoei et al. using primary cholangiocarcinoma cells, cyclophilin A (CyPA), a ligand to CD147 was shown to bind on CD147 and stimulate extracellular receptor kinase 1/2 (ERK1/2) phosphorylation followed by upregulation and phosphorylation of cyclin D1 and retinoblastoma protein (RB), leading to G1/S transitioning thus the propagation of cell cycle (54). The integral role of CD147 in the cell cycle is again evident in the study by Kendrick, A. A. et al. in which knockdown of CD147 by shCD147 in pancreatic cancer PANC1 cell line led to S/G2 cell-cycle arrest, a phenomenon that co-occurred with depletion of glycolytic intermediates that act as building blocks in the cell cycle process (45). Notably, the trafficking of glycolytic intermediates is also mediated by CD147 which acts as an indispensable symporter to the monocarboxylate transporters (MCTs), a role discussed under the ‘Warburg Effect’ section (43).

Underlying cancerous cellular division and multiplication processes is the stemness characteristic found in subpopulations of cells existing in almost all types of cancer (55). Whether pre-existing cancer stem cells (CSCs) or as derived from differentiated cancer cells (cancer cell plasticity), these CSCs are known to be the key drivers of cancer development, progression, and adaptation (56). Several molecules and pathways are involved in the functional activation or induction of CSCs, among these are transcriptional factors such as signal transducer and activator of transcription (STATs), Sox, Oct4, and NANOG proteins (57). Interestingly, CD147 potentiates and regulates the stemness of several cancer cells through these molecules: in the study by Meng, Y. et al. using breast cancer cells, detachment of the MDA-MB-231 cells resulted in CD147-mediated potentiation of non-CSCs into CSC characteristics that were featured by resistances to anoikisis, sphere formation, and expressions stem cell markers such as CD44 (35). In that study, CD147 was shown to regulate stemness induction through the CD147-CyPA-STAT3/Bcl-xL signaling pathway. Notably, downregulation of CD147 shCD147 lentiviral transfection rendered the cells anoikisis sensitive and unable to emanate stemness characteristics (35). In another study by Li, L. et al. stemness-induced malignancy and resistance to chemo-radio-resistance found in pancreatic cancer cells could be reversed through inhibition of CD147 activity using the antibody cHAb18 (44). This CD147 arrest affected the CDs44-STAT3 pathway, reduced the expression of transcription factors NANOG, OCT4, and SOX4, and rendered the cells sensitive to gemcitabine-cHAb18 combined therapy (44).

Regarding cellular proliferation in human cancer cells, Kras-mediated signaling is one of the major players in proliferative signal transduction (58). In lung cancer cells for example, proliferation is regulated through the Kras–Erk–NF-kB–Timp1–CD63–FAK–Erk loop: activation of the cellular proliferation process through this pathway requires Timp1 and its receptor CD63, and Timp1 is the target gene for NF-kB (59). NF-kB and Timp1 (tissue inhibitor of metalloproteinase 1) are usually overexpressed in advanced lung cancer, and they are associated with poor prognosis (59). Interestingly, CD147 upregulates the molecular expression and function of NF-kB (60); in the study of head and neck squamous cell carcinoma (HNSCC) by Yu et al, the CD147 gene was silenced through lentiviral transfection of HN4 and HN30 cancer cells with CD147-Homo-550 (siRNA) (shCD147) with shNC as controls. In comparison to their controls (shNC), shCD147 cells had significantly reduced expression of markers for NF-kB activation (p-IKKα, p-IκBα, p-p65) even after induction with TNFα. These findings were associated with CD147-dependent regulation of cellular proliferation and evasion of apoptosis *in vitro*, as well as tumor initiation and growth *in vivo* (34). In glioma cell line U251, downregulation of CD147 expression by small interfering RNA (siRNA) resulted in suppressed cell proliferation, cell cycle arrest, and induction of apoptosis (61). CD147 is, therefore, an imperatively essential molecule in the neoplastic cell cycle, proliferation, and stemness acquisition, hence a hotspot target to abrogate the proliferation and growth potential of cancers.

### 2.2 CD147 “Switches on” angiogenesis and metabolic rewiring in cancers

Rapid growth, high metabolic demand, immune response, and genetic alterations trigger tumors to derail from the balanced pro-angiogenic and anti-angiogenic balance, switching into explosive angiogenesis by recruiting extensive networks of new blood vessels. Tumor angiogenesis occurs by either sprouting in which the parent vessel buds off sprouts or bridges into the tumor tissue; intussusception in which interstitial cells block the vascular lumen of the pre-existing vessels thus inducing remodeling and expansion; or vasculogenesis involves recruitment of bone marrow- or peripheral blood-derived endothelial precursors to form endothelial lining of tumoral vessels. through block, sprouting, or incorporation of the endothelial precursors (62). These processes involve an interplay of molecules from different origins including tumoral cells, endothelial and stromal cells, as well as blood and extracellular matrix (62). There are numerous pro-angiogenic molecules involved in angiogenic signaling in cancers, to mention a few are vascular endothelial growth factor (VEGF) which stimulates angiogenesis, vasculogenesis, vascular permeability, and cellular adhesion; matrix metalloproteinases (MMPs) and plasminogen activators crucial for matrix remodeling, release, and activation of growth factors; VEGF receptors (VEGFR) that integrate signals for angiogenesis and survival, as well as fibroblast growth factors (FGF), Tumor Growth Factors (TGF) and platelet-derived growth factors (PDGF) (62). Despite the robust mechanisms by which cancers recruit new blood vessels, their microenvironments remain predominantly hypoxic, low pH, and nutrient-deprived; this results from the fact that the newly formed vessels are usually defective both structurally and functionally (63). To parallel this, cancers develop adaptive mechanisms to cope with the constantly hypoxic, acidic, and under-nourished environment meanwhile maintaining a high rate of cellular proliferation and dimensional growth. CD147, directly or through signal cascades to downstream molecules plays an integral role in both angiogenesis and hypoxic and metabolic adaptation exhibited by cancers as discussed in the next paragraphs.

**Figure 1:**
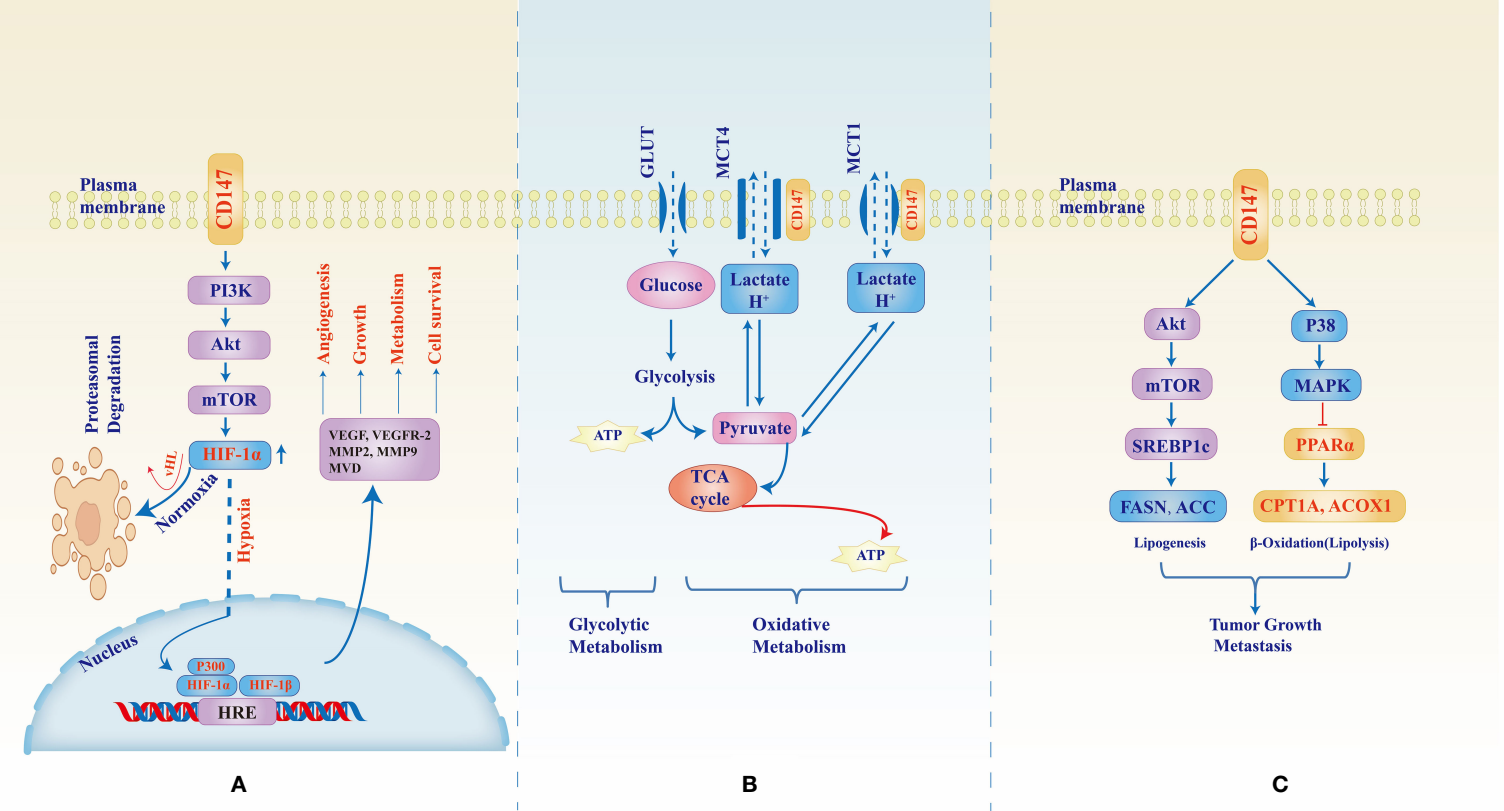
CD147-mediated signaling pathways in tumor development, growth, metastasis, and metabolic reprogramming. CD147 upregulates expression of HIF-1α through the PI3K/Akt/mTOR pathway **(A)** however, under normal oxygen supply, HIF-1α undergoes Von Hippel–Lindau (vHL) factor mediates hydroxylation and ubiquityl proteasomal degradation. In hypoxic conditions, the cytoplasmic molecule stabilizes, increases, and translocates to the nucleus where they dimerize with HIF-1β and induce transcription of hypoxia response element (HRE) which in turn enhances expression of factors necessary for angiogenesis, tumor growth, and adaptive metabolism. Figure **(B)** shows the role of CD147 as a chaperone to monocarboxylate transporters (MCTs) 1 and 4. Due to the unmatched supply of oxygen, rapidly growing tumors rely largely on lactate oxidative metabolism for energy production and therefore CD147-MCT1/4 interaction becomes the backbone to sustain lactate and H+ trafficking and pH balance. Figure **(C)** shows the CD147-mediated increase in fatty acid synthesis necessary for tumor growth machinery whereby CD147 through Akt/mTOR/SREBP1c pathway activates the enzymes responsible for lipogenesis while at the same time inhibiting β-oxidative degradation of lipids through p38/MAPK activation which inhibits PPARα, an activator of the degrading enzymes.

To evade the deleterious consequences of hypoxia, cancer cells activate the repertoire of pro-angiogenic, and anti-angiogenic molecules as discussed above, by upregulating synthesis and expression of hypoxia-inducible factors (HIFs), a group of helix-loop-helix heterodimers which masters the cellular adaptation to mitigate the constant oxygen, nutrients, and excretory gap (64). The two cytoplasmic subunits HIF-1α and HIF-2α, are the principal regulators of adaptation with their common counterpart subunit HIF-1β which is constantly localized in the nuclear. HIF-1α and HIF-2α activities are complimentary but nonoverlapping, HIF-1α undergo rapid stabilization and activation under acute and severe hypoxic condition meanwhile HIF-2α has a gradual activation under moderate and chronic Hypoxia; stabilization HIF-1α and consequent nuclear dimerization with HIF-1β induces transcription of anaerobic glycolysis and cell death regulator genes, that of HIF-2α induces transcription of erythropoietin synthesis, stemness, and pluripotency regulator genes (65). In the upstream, CD147 regulates the expression HIF-1α and HIF-2α, thus one of the “main switches” of angiogenesis, hypoxic and metabolic rewiring of cancers (38, 66, 67). *In vitro* and *in vivo* study of melanoma WM278 and M10 cell lines by Bougatef F. et al, physiological and induced overexpression of CD147 translated into upregulated expression cytoplasmic HIF-2α and nuclear HIF-2α/HIF-1β nuclear dimers. Under hypoxic conditions, oxygen-dependent hydroxylation and ubiquityl-degradation of HIF-2α is halted therefore, HIF-2α accumulates in the cytoplasm and can translocate to the nuclear, dimerize with HIF-1β, and mediated VEGFR-2 expression, cellular proliferation, diminished apoptosis, and angiogenesis (66) (**Figure 1A**). In the same study, CD147 expression also induced the expression of MMP-2 and urokinase-type plasminogen activator (uPA), molecules involved in the degradation of the extracellular matrix to enable tissue and vascular growth. Immunohistochemical analysis of gastric carcinoma tissues showed that CD147 expression correlated with the expression of pro-angiogenic molecules such as VEGF, micro-vessel density (MVD), MMP-2, and MMP-9 (68).

PI3K/Akt/mTOR signaling pathway has been extensively studied and reported to be one of the principal signaling cascades in normal and malignant tissues; aberrant or hijacked PI3K/Akt/mTOR modulation is associated with cancer development, adaptation, and progression (69). CD147 is one of the upstream regulators for the PI3K/Akt/mTOR pathway: using prostate cancer PC-3 cells, Fang F. et al. showed that CD147 through PI3K/Akt/mTOR pathway could rescue the cancer cells from, autophagy under induced starvation (50). The cells were cultured in an amino acid-free EBSS medium, found to induce a gradual increase of CD147 expression consequent phosphorylation of Akt and mTOR was upregulated thus PI3K/Akt/mTOR activation that was shown to inhibit autophagy through inhibition of autophagosome formation as marked by the LC3 protein; silencing of CD147 by shCD147 plasmid resulted into decreased p-Akt and p-mTOR and subsequent increase of autophagosome formation and enhanced cell death (50). In another study by Fei F. et al. who used immunohistochemical staining of non-small cell lung cancer (NSCLC) tissues from human patients as well as *in vitro* study of NSCLC A549 cell lines, CD147 was shown to form a complex with CD98, a cell-membrane heavy chain-light chain heterodimer known to interact with other molecules such as integrins and influence cellular proliferation, migration, and survival (37). CD147 and CD98 had correlational expression and their co-immunoprecipitation was associated with poor prognosis and poor overall survival; CD147-CD98 was shown to activate PI3K/Akt pathway which resulted in increased proliferation and tumor growth as marked by the mitotic index and Ki-67 (37). We have stressed in this section the key role of hypoxia-inducible factors 1/2 in circulation and metabolic reprogramming witnessed in cancers and that of CD147 as their upstream regulator. CD147 overexpression under hypoxic conditions regulates that of HIF-1α downstream; growth and survival under hypoxia of hepatocellular carcinoma SMMC-7721, HCC-9204, and LX-2 cell lines were shown to be affected by CD147 and HIF-1α expressions whereby hypoxic exposure of HCC-9204, and LX-2 yielded a time-dependent upregulation of HIF-1α which also correlated with that of CD147 (38). However, CD147 expression was relatively lower in LX-2 compared to other cell lines and thus thought to result in reduced viability of the LX-2 cells under hypoxia (38). Further exploration of CD147 and HIF-1α interaction and angiogenesis can be observed in another study by Wang C. H. et al, using rheumatoid arthritis patients’ tissues, fibroblast-like synoviocyte (RA FLS) cell cultures, and SCD mice engrafting, CD147 expression correlated with that of HIF-1α, VEGF, vascular endothelial cells, and vascular density in stained tissues, and that in RA FLS cells upregulated expression and secretion of HIF-1α and VEGF; silencing of CD147 with siCD147 or anti-CD147 monoclonal antibody (infliximab) resulted into a significant decrease of HIF-1α and VEGF expressions as measured by real-time PCR, ELISA, and western blotting; vascular density was also reduced in mice treated with anti-CD147 (67). Further analysis revealed that CD147 upregulated the expression of HIF-1α and VEGF through the PI3K/Akt signaling pathway. PI3K/Akt/mTOR signaling pathway is therefore one of the major alternate pathways through which upstream regulators such as CD147 modulate HIF-1α expression and function resulting in adaptive angiogenesis and metabolic reprogramming exhibited by cancers (65, 70, 71).

#### 2.2.1 CD147 and the Warburg effect

The 1926 reports by Otto Warburg et al. about carbohydrate metabolism of sarcomas and carcinomas postulated the glycolytic switch of cancers by increasing glucose uptake by multiple-folds from which they split glucose to generate energy (ATP) and lactate even in the presence of oxygen (fermentation or aerobic glycolysis), later suggesting a dysfunctional mitochondrial metabolism as normal cells would generate ATP, water, and CO_2_ under aerobic catabolism of glucose (72). Follow-up studies have continued to modify such concepts with variations about the reason for such a metabolic switch. However, although some tumors exhibit a substantial magnitude of respiration as initially reported by Crabtree H. in 1929 (73), up to date it remains clear that aerobic glycolysis is the predominant powerhouse for tumors’ growth and survival, and the rate of aerobic glycolysis is 10 to 100 folds that of mitochondrial respiration (74). In addition to that, increased glucose uptake provides building blocks for proteins, lipids, and nucleotides biosynthesis (75); rapid glucose uptake and lactate efflux alter the tumor microenvironment by draining glucose away and lowering extracellular pH which in turn diminishes the immune capability of tumor-infiltrating lymphocytes (TILs) and enhances invasiveness; also, aerobic glycolysis indirectly induces cell signaling modulation to maintain reactive oxygen species homeostasis and acetylation-deacetylation cellular activities (74). Integral to the aerobic glycolysis (Warburg effect) of cancers are the transmembrane trafficking of glucose and lactate activities to which CD147 is at the core of the glucose and monocarboxylate transporting machinery:

CD147 is both a trafficking chaperone and regulator to lactate transporters, monocarboxylate transporter 1 and 4 (MCT1/4) which is crucial for lactic glycolysis by cancers in normoxia and hypoxia (**Figure 1B**). Electronic microscopic analysis of CD147-MCT interaction showed that CD147 single transmembrane domain interacted with the 6^th^ among the 12 transmembrane domains of MCT1; the conformational CD147-MCT1 interaction enables the inward and outward opening of MCT1 with resulting shuttling of lactate (43). In another *in vitro* study of pancreatic ductal adenocarcinoma (PDAC), CD147 had a striking influence on several cancer metabolic activities such as lactate co-transportation with MCT1 and MCT4, regulated phenotypic expression of MCT1 and MCT4, and also the regulation of key metabolic enzymes including aldehyde dehydrogenase 1 A3, transglutaminase 2, pyruvate kinase isoenzyme 2, and glucose-6-phosphate dehydrogenase (45). Loss of function or depletion of CD147 has been shown to have a detrimental effect on overall cancer growth and survival. Treatment with shCD147 to PDAC cells led to downregulation of MCT1, MCT4, and several metabolic enzymes, and accumulation of intracellular lactate resulting in halted cellular proliferation, adhesion, and migration (45). Baba M. et al. Disrupted the CD147-MCT1 interaction using anti-CD147 monoclonal antibody (MEM-M6/1) in colon cancer, melanoma cells, and normal fibroblasts; the inhibition resulted in failure of lactate shuttling, ATP depletion, decreased cellular pH and cell death of the cancer cells with no similar effect in normal fibroblastic cell (47), this affirmed the dependence of cancer cell on lactate glycolysis for growth and survival. Inhibition of CD147-MCT4 conformational interaction by Acriflavine molecule in primary glioma stem cells led to reduced hypoxia-induced transcription of HIF-1α, lactate export, and inhibition of tumor growth and vascularization (76). By function of its transmembrane domain, CD147 also acts as an ancillary protein to the MCTs by protecting them from proteasome-degradation and orchestrating their cytoplasm-to-membrane translocation (45). Therefore, CD147 is not only integral but also essential and critical in aerobic glycolysis thus the growth and survival of cancer cells.

Apart from its critical role in the carbohydrate metabolism of cancers, CD147 has also been shown to be essential in both fatty acid and amino acid metabolism. Data analysis from the TCGA and Gene Expression Omnibus (GEO) for the expression of CD147 and genes responsible for aberrant fatty acid metabolism in HCC, as well as *in vivo* and *in vitro* studies of SMMC-7721 and MHCC97L cell lines showed that CD147 correlated with, and upregulated sterol regulatory element binding protein 1c (SREBP1c) expression through Akt/mTOR pathway; SREBP1c then activates FASN and ACC1 gene transcription with resultant *de novo* fatty acid synthesis. CD147 also inhibited fatty acid degradation through p38/MAPK activation which in turn downregulates PPARα and target genes ACOX1 and CPT1A, involved in β-oxidation of fatty acids (41) (**Figure 1C**). CD147 overexpression in pancreatic cancer correlated with that of amino acid (AA) transporters, L-type AA transporter (LAT1, an mTOR mediated essential AA transporter), system ASC AA transporter-2 (ASCT2, a neutral AA and glutamine transporter), and the heavy chain of LAT1 (4F2c, known to interact with CD147-MCT complex as well as LAT1) (24, 45). Co-overexpression of CD147 with these AA transporters was associated with tumor growth, metastasis, and worse prognosis in cancers (24, 77, 78).

### 2.3 CD147 enables invasiveness and metastasis of cancers

CD147 has been known for its role as an upstream regulator for the expression of molecules responsible for extracellular matrix modification (matrix metalloproteinases, MMPs) such as MMP-2, MMP-3, MMP-9, hence the name matrix metalloproteinase inducer, EMMPRIN (79, 80). CD147 induces vesicle secretion of MMPs by the malignant cells meanwhile the receptors for the molecules are located on surrounding normal fibroblasts and other stromal cells (16). The binding of MMPs on the stromal cells stimulates matrix breakdown, endothelial-mesenchymal transitioning (EMT), and increased vascular permeability, conditions necessary for tumor invasiveness and metastasis of tumors; inhibition of CD147 by knockdown, siRNA, shRNA, or monoclonal antibody in several aggressive human cancers such as U251 glioblastoma and T7721 hepatoma cell lines led to reduced cell adhesion, invasiveness, migration, and metastasis (39, 81). In the study using retinoblastoma tissues and SO-RB50 and RB-Y79 cell lines by Wu Z. et al, upregulation of microRNA-4319 whose target is to inhibit CD147 resulted in inhibited cellular proliferation, migration, and EMT progress in basal or silenced CD147 while overexpression of CD147 restored these properties (40). Notably, it is during the EMT process that tumor cells become very potent to undergo migration, invasion, and metastasis (82).

## 3 CD147 functional role in cancer-enabling characteristics

### 3.1 DNA damage response

Cancers are known to acquire self-regulatory responses to DNA damage that elude the intricate and interlocking normal cellular mechanisms of DNA damage response (DDR). Radiations, for example, inflict DNA damage as single-strand breaks (SSBs) or double-strand breaks (DSBs). Normally, SSBs are repaired through base excision repair or nucleotide excision repair. Homologous recombination and non-homologous end-joining (NHEJ) are methods of repair that cells deploy in DSB-damage; usually, homologous recombination is a conservative process aiming at restoring the original DNA sequence before damage and it occurs in the S-phase and G2-phase of the cell cycle under the mediation of cyclin and cyclin-dependent kinases (CDKs) (83). Therapeutic radiations target to exploit DDR in malignant cells by inducing DSBs that would result in cell cycle arrest at the G2/M phase and apoptosis. In contrast, NHEJ tends to directly ligate the DNA segment distal to DSB, and this can occur at any given point of the cell cycle (84). DDR pathways in tumor cells do not adhere to the strictly coordinated process of DNA damage detection, cell cycle arrest and accumulation of repair factors, and physical repair that are meant to ensure the maintenance of genomic integrity; as a result, this violation guarantees suboptimal repair and propagation of aberrant DNA strands (85). Expression of CD147 in cancers is associated with aberrant DDR; in the analysis of pancreatic ductal adenocarcinoma patients from The Cancer Genome Atlas (TCGA) database, CD147 had a significant positive correlation with DDR footprint indices most of which (10/16) indicate altered DNA copies. Interestingly, the altered DNA copy-indices that were associated with high CD147 expression had fold change (FC) of above five; high CD147 expression was again significantly associated with a higher percentage of arm-level gain rather than arm-level loss among the 22 pairs of autosomal chromosomes (p<0.0001), thus CD147 expression contributes to genomic instability and propagation of pancreatic adenocarcinoma (86). By inducing DNA damage through treating pancreatic cancer CFPAC-1 cells with gemcitabine, an anti-cancer drug that disrupts DNA synthesis by introducing a mismatching 2′,2′-difluoro-2′-deoxycytidine triphosphate (dFdCTP) nucleoside into the DNA sequence, CD147 rescued the cancer cells from undergoing apoptosis by inducing phosphorylation of p53 through the ATM/ATR/p53 pathway; phosphorylated p53 is stable from Mdm2-mediated ubiquityl degradation therefore, p53 translocates to the nuclear and activates DNA repair (86). γH2AX is a protein marker for G2/M phase-homologous recombination DSBs repair; *in vitro* and *in vivo* studies, induction of DSBs through treatment with radiations of cervical cancer SiHa and ME-180 cell lines resulted in CD147-dependent evasion of DDR and cell cycle rescue; exposure to 10-Gy of CD147^+^ SiHa and ME-180 had a rapid decrease of γH2AX within 1 and 6 hours of exposure respectively meanwhile CD147^-^ cells had a rapid increase of γH2AX, reaching maximal values within 30 minutes of exposure and remained at maximal for 24 hours post-exposure. On cell cycle regulation, CD147 was denoted as responsible for limiting the number of cells in the G2/M phase (DSBs repair point) whereby CD147^-^ cells had a significantly increased percentage of cells in the G2/M phase as compared to CD147^+^ cells (48).

### 3.2 Mediation of immune manipulation in tumor microenvironments

Cancers are known to effectively suppress the immune response through the activation of several pathways that mount a negative regulatory effect on the immune response. CD147 plays direct and indirect roles in this immune evasion by cancer cells. As the backbone to the metabolic switch, glucose depletion and lactate-dependent glycolysis derived from CD147-MCTs interaction lead to tumor-infiltrating lymphocytes (TIL) dysfunction following reduced glucose uptake and reduced GLUT1 expression (87). CD147-MCT-mediated lactate efflux leads to extracellular acidosis which in turn, directly inhibits natural killer cells’ cytotoxicity as well as increases the number of myeloid-derived suppressor cells (87). In the *in vitro* and *in vivo* mice study by Chen Y. et al. on B16-F10 melanoma and Lewis lung cancer cell lines, there was upregulation of CD147 on the cell membrane of TILs which also co-expressed with Tim-3 and PD-1, known immune checkpoint molecules. CD147 knockout was associated with an increase in TILs, CD8^+^ cytotoxicity, PD-1^+^ CD8^+^, cytotoxic molecules such as perforin, TNF-α, and INF-γ, and reversed PD-1^+^Tim-3^+^CD8^+^ TILs dysfunction (88).

## 4 Discussion

**Figure 2:**
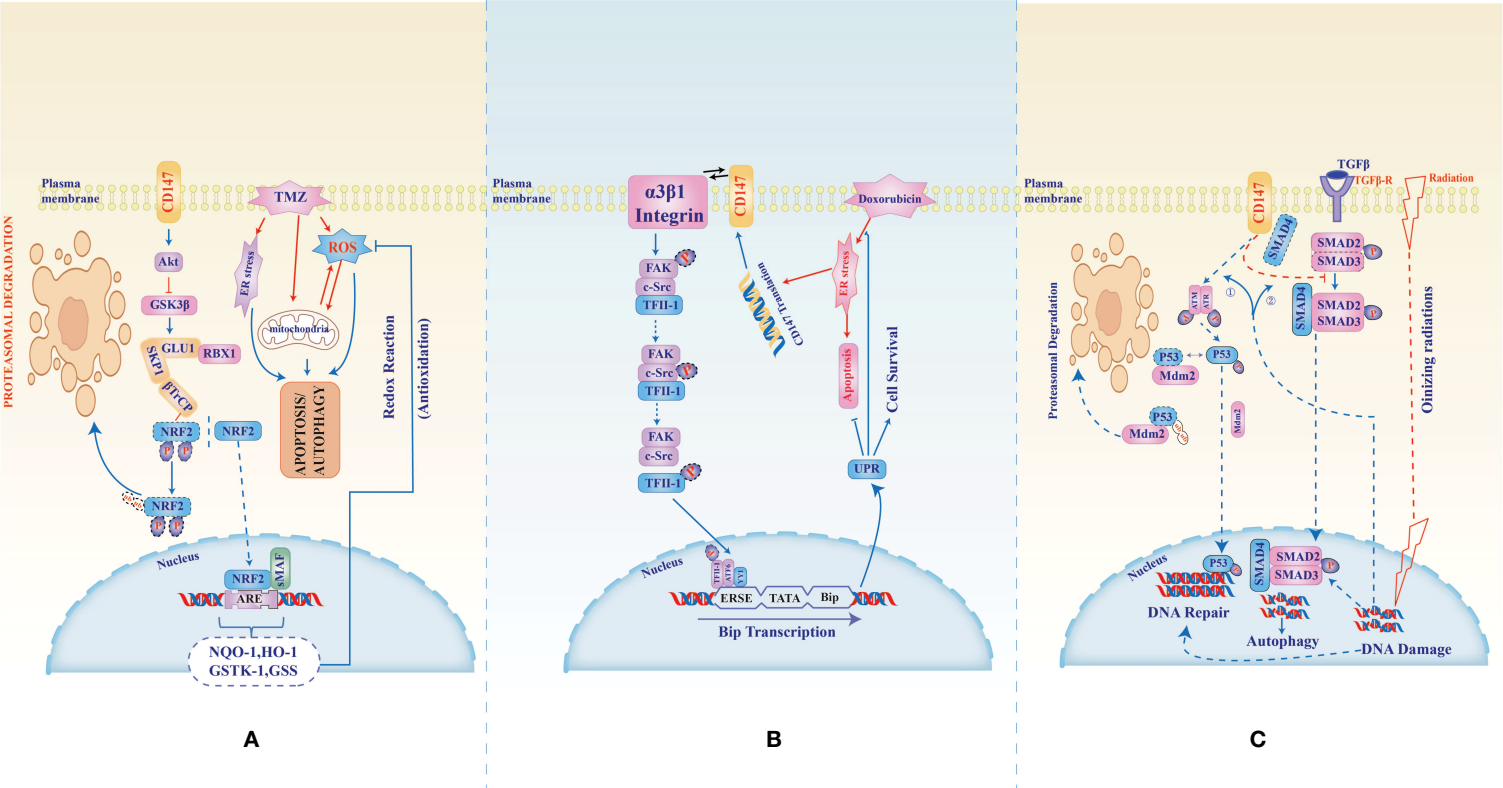
CD147-mediated multidrug resistance in glioblastoma, hepatocellular carcinoma, Ovarian, and pancreatic cancer. In the treatment of Glioblastoma with Temozolomide, an anti-cancer agent known to induce reactive oxygen and endoplasmic stress as well as mitochondrial damage **(A)**, CD147 counteracts the cytotoxicity through inhibition of phosphorylation hence stabilization of NRF2 (nuclear factor erythroid-related factor 2). Stabilized NRF2 translocates to the nucleus and activates the transcription of genes (antioxidant response elements, ARE) responsible for anti-oxidative cellular protection. **(B)**, CD147-α3β1-integrin interaction activates phosphorylation of transcription factor II-1 which together with ATF6 and YY1 binds ERSE to activate Bip protein transcription which in turn activates the unfolded protein response that inhibits the cytotoxic effect of doxorubicin in hepatocellular carcinoma. In **(C)**, the cellular fate from DNA damage resulting from ionizing radiations or gemcitabine chemotherapy in ovarian and pancreatic cancers is altered by CD147 which induces phosphorylation and stabilization of p53 through ATM/ATR. Phosphorylated p53 translocates to the nuclear to mediate DNA repair hence evasion of DN damage-induced cell death. Also, CD147 binds SMD4, switching off the signal from TGFβ to which SMD4 is necessary to induce an autophagic response to radiation-induced reactive oxygen stress.

Heuristic conceptualization of cancer disease into key features named hallmarks of cancer and cancer-enabling characteristics provided an additional roadmap for deciphering the complex and aberrant genotypic and phenotypic cancer manifestations. These features of cancer manifestation are known to be distinct and yet overlapping which in turn presents an avalanche of evasive and transformative custodianship of roles, this in turn presents uncertainty in identifying hotspot targets for developing effective and safe cancer treatments. In that light, determining a molecule common across heterotypic cancer types, cancer characteristics, and neoplastic signal transductions would provide a viable hotspot with an explosive anti-cancer effect. Apart from being involved in cancer development, progression, metastasis, and recurrence, CD147 has also been associated with resistance to multiple anti-cancer therapies such as Temozolomide (89), Cisplatin (90), Oxaliplatin (91), Biguanides (Metformin) (92), Doxorubicin (93), Ara-C (Cytarabine) (80), Gemcitabine (86), Paclitaxel (94), Epirubicin (95), Docetaxel (96), Vemurafenib (97), and Radiotherapy (48); exhibited in gliomas, carcinomas of the Larynx, lung, breast, gastric, liver, pancreas, colon, bladder, prostate, ovary, and cervical; melanoma, and lymphomas (**Figure 2**). Nevertheless, inhibition of CD147 activities in these studies rendered the cancer cells sensitive to the treatments. In that light and considering its near-universal role in the acquisition and propagation of hallmarks of cancer and cancer-enabling characteristics in most aggressive human cancers, CD147 is inevitably a viable and potential target molecule by both conventional and targeted therapies.

### 4.1 Enhancing efficacy and safety with CD147-targeted therapies and immunotherapies: current and future clinical applications

**Figure 3:**
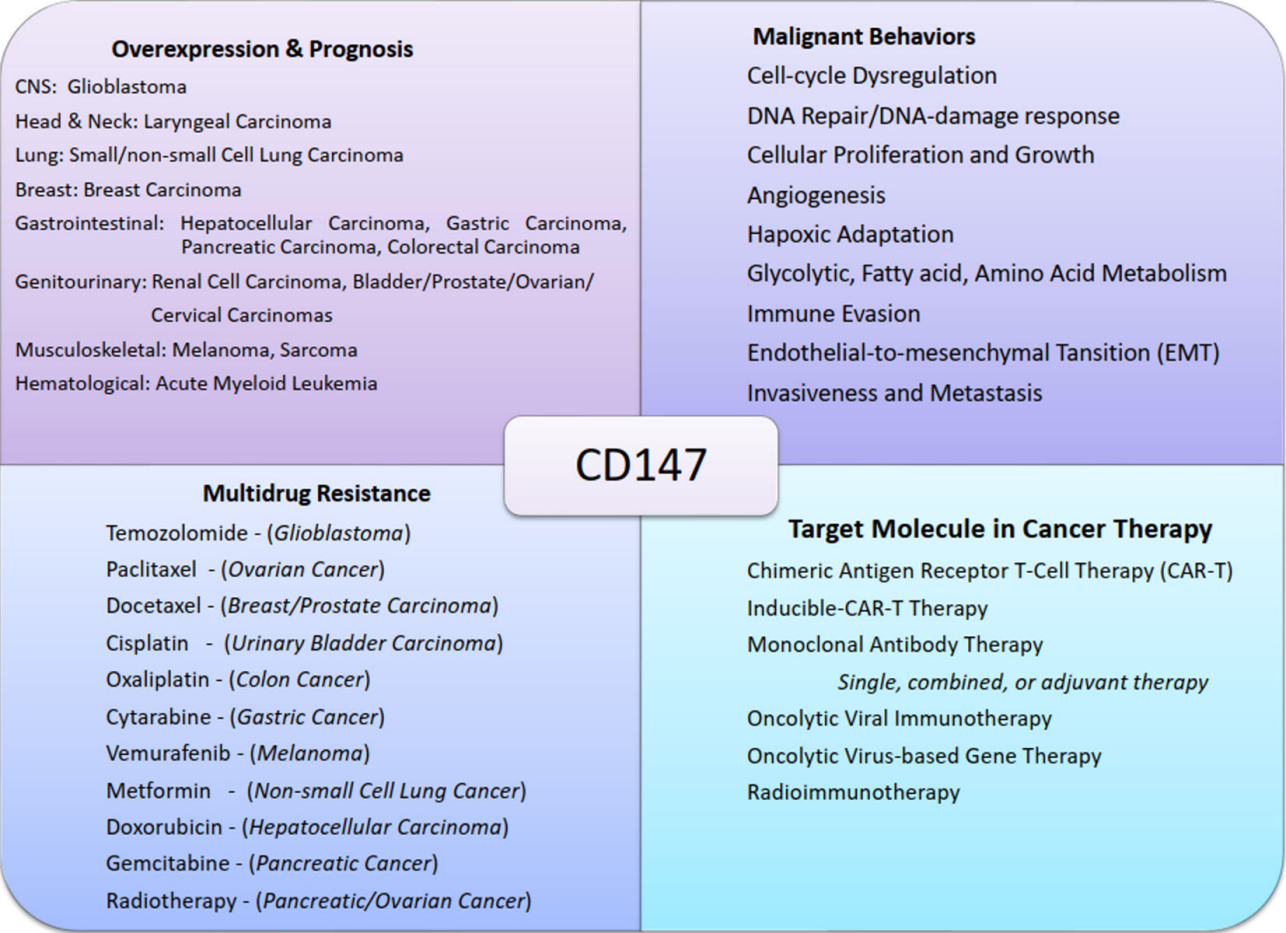
Chart summary of near-universal involvement of CD147 in cancers. The figure shows examples of malignant tumors of different systems that express CD147, its role in cancer progression, CD147-mediated multidrug resistance, and the potential therapeutic designs exploiting CD147 functions.

Therapeutics targeting CD147 are more likely to be successful because the molecule is involved in numerous pro-oncogenic and oncogenic molecules and pathways, such as MMPs (18), MCTs, GLUTs, and MDRs (96, 98); CyPA (54), Lewis y antigen (29), FUT1 (42), NDUFS6 (99), ERK-1/2 (46), and PI3K/Akt/mTOR (50). The potential therapeutics targeting CD147 include chimeric antigen receptor T cell (CAR-T) therapy (100), inducible CAR-T therapy (101), antibody therapy (102), chimeric antibody and intrabody therapy (103, 104), and radioimmunotherapy (RIT) (105). CD147 can also be targeted in adjuvant or combined therapies such as combined oncolytic viral, gene, and antibody therapies (106, 107), combined monoclonal antibody and radiotherapies (108), adjuvant monoclonal or chimeric antibody therapies (109, 110), and adjuvant radioimmunotherapies (110) (**Figure 3**; **Table 2**).

**Table 2:** Targeted Cancer Therapeutic Designs Against CD147.

Targeted Therapy	CD147 Application in the Design	Therapeutic Outcomes	Current Application	References
Chimeric Antigen Receptor T cell (CAR-T) Therapy	CD147-single chain fragment variable (scFv) targeted by T and NK cells (CD147CAR)	Enhanced cytotoxicity, Reduced off-target toxicity	Experimental studies (Hepatocellular Carcinoma)	(100)
Inducible CAR-T therapy	Conjugated CD147CAR to Tet-On 3G system and Doxycycline ((Dox+) Tet-CD147CART)	Increased cytotoxic activity to targets Reduced toxicity and adverse events	Experimental studies (Hepatocellular Carcinoma)	(101)
Antibody Therapy	Recombinant CD147 antibody to induce antibody-dependent cellular cytotoxicity (ADCC)	Increased Efficacy and Reduced toxicity	Phase I Randomized Clinical Trial (NSCLC)	(111)
Chimeric Antibody Therapy and Radioimmunotherapy	Coupling CD147/HAb18 with radioactive iodine 131 (I^131^-Metuximab)	Increased relapse-free survival (RFS) Reduce grade 3, and 4 adverse events	Phase II Randomized Clinical Trial (Hepatocellular carcinoma)	(103, 105)
Chimeric Intrabody Therapy	Chimeric Adenovirus vector with single chain fragment variable (scFv-M6-1B9) that targets CD147 to induce cytoplasmic retention of CD147	Suppressed invasiveness of invasive cervical cancer	Experimental studies (Cervical Cancer)	(104)
Antibody-Drug Conjugate (ADC)	CD147 antibody (HcHab18) conjugated to Emtansine (DM1)	6-times increase in cytotoxicity than HcHAb18 alone	Experimental studies (NSCLCs)	(112)
Combined Gene Therapy	CD147/HAb18 conjugated to Adenovirus vector carrying target genes	Increased tumor tropism and enhanced cytotoxicity	Experimental studies (Hepatocellular carcinoma)	(107)
Combined Oncolytic Viral Therapy	Conjugation of CD147/Hab18 to Newcastle Disease Virus or Adenovirus	Enhanced cytotoxicity, arrested metastasis	Experimental studies ((Hepatocellular Carcinoma)	(106)

In hepatocellular carcinoma, CD147-CAR-modified T and NK cells targeting the single chain fragment variable (scFv) domain of CD147 increasingly expressed in HepG2 and SK-Hep1 cell lines were shown to have high cytotoxicity specific for CD147^+^ HCC cells, *in vitro* and *in vivo* (100). The therapy had reduced cytotoxicity in cells expressing low CD147 irrespective of the effector-target ratio. Inducible CAR-T therapies with the duality of CD147 and additional tumor-specific antigens or other molecules further increase tumor-specific cytotoxicity and reduce on-target and off-target toxicity (100, 101). Creation of GPC3-synNotch-inducible CD147-CAR was shown to have higher cytotoxic activity on HCC cells expressing CD147^+^GPC3^high^ with no activity against CD147^-^GPC3^high^, CD147^+^GPC3^low^, or healthy human tissue (100). Also, designed doxycycline-inducible lentiviral-coded CD147-CAR regulated by the Tet-On 3G system (LV-Tet-CD147CAR-T), had a doxycycline-dependent increased proliferative and cytotoxic activity of CD4^+^ and CD8^+^ T cells against CD147^+^ HCC cells, both *in vitro* and *in vivo* (101).

In antibody-antigen targeted therapies, CD147 could be an effective target molecule in both monoclonal antibodies, chimeric antibodies, antibody-drug conjugate (ADCs), and radioimmunotherapy therapeutic designs. Again, tumor-specific expression of CD147 renders its corresponding antibody potential for incorporation in both oncolytic viral and gene therapies.

Development of the chimeric monoclonal antibody HAb18 from BALB/c mice immunized with CD147^+^ human HCC tissue showed a 99.55% binding rate against human antigen CD147/HAb18G with evident FAK-PI3K-Akt-Girdin pathway-mediated tumor-specific cytotoxicity (103, 105). Coupling the mA HAb18 with radioactive iodine 131 through pepsin-digestion removal of the Fc fragment of HAb18 to form I^131^mA HAb18 (I^131^-Metuximab), had a radiation absorption of 2.5 to 18.6 times that of nontumor tissue in an animal study, improvement of hematologic indices in 62.5% during phase I clinical trial, and during phase II trial had grade 3 hematologic toxicity in only 1 out of 14 (7.14%) and 4 out of 89 patients (4.49%) during preliminary and trial period respectively, with only <10% aggravated hepatic damage (105). Phase II randomized control trial using I^131^mA HAb18 adjuvant to curative-resection involving 156 eligible patients had a 5-year relapse-free survival (RFS) of 43.4% versus 21.7% in the control group, with a hazard ratio of 0.49, *p=0.0031*, and only seven out of 78 treatment group patients had grade 3 or 4 adverse events (110).

Metuzumab is another CD147-antibody dependent cellular cytotoxic (ADCC) agent, developed by recombination and glycoengineering of human and mouse chimeric immunoglobulin G1 monoclonal antibody against CD147 extracellular C2 domain; it is non-fucosylated and therefore, has optimized affinity to the Fc fragment subtype FcγRIIIa, which in turn increases the affinity to ADCC promoters such as NK cells (102, 111). This mA HAb18 modification enhances the cytotoxic potency of the CD147 monoclonal antibody by 10 to 20 times in HCC cells with significant *in vivo* tumor growth arrest (p<0.01) and was well tolerated without adverse events in animal studies (111).

ADC designed using maytansinoid-based cytotoxic agent emtansine (DM1) linked by the non-cleavable chemical linker SMCC to CD147 antibody HcHAb18 (HcHAb18-DM1) in NSCLC cells both *in vitro* and *in vivo*, was shown to be specifically internalized by CD147^+^ tumor cells, successfully delivering the payload (DM1) and releasing the active metabolites through lysosomal cleavage, leading to cell cycle arrest at G2/M (112, 113). This activity is dependent on CD147 expression and high proliferative cells, features significantly in malignant cells than in normal tissue cells; HcHAb18-DM1 was six times more effective than HcHAb18 alone and no activity with DM1 alone either, and there was equal weight gain in both treatment and control animal study groups (112).

In oncolytic viral immunotherapy tumors are genetically engineered by being infected with attenuated viruses to enhance tumor tropism which in turn enhances antigen recognition or release consequently counteracting immune evasion of tumors through immune reactivation (114–116); one of the greatest challenges in employing such viruses like Adenovirus, Herpes Simplex Virus, vascular stomatitis virus, vaccinia, and Newcastle disease virus, is the failure of sufficient drug delivery to the tumor which can therefore be resolved by conjugation with cHAB18G, whose target antigen CD147/HAb18 is near-universally and differentially expressed in tumor tissues (106, 114–119). Survivin promoter-regulated oncolytic adenovirus vector carrying an anti-oncogenic gene P53 (AdSurp-P53) have significant cytotoxic activity than Ad-P53 *in vitro* and *in vivo*, gallbladder cancer EH-GB1 cell line (120), and Adenoviral vector carrying an anti-oncogenic human sulfatase 1(hSulf-1) gene (Ad5-hSulf1) have anti-phosphorylation mediated anti-proliferative and anti-angiogenic activity against ovarian and hepatic cancer cells (107); therefore engineering a recombination of the two oncolytic virus-based gene therapies AdSurp-P53 and Ad5-hSulf1 with I^131^mA HAb18 through addition and replacement of the CArG element of early growth response-1 (Egr-1) and P53 respectively from AdSup-P53 to form AdeSurp-hSulf1- I^131^mA HAb18 combined therapy, resulted into an even more effective tumor-specific cytotoxic activity against HCC cells, a very potential CD147 based combined therapy (radioimmunotherapy, oncolytic therapy, and gene therapy) (117).

In the study by Ding W. et al, the insertion of light and heavy genes coding for CD147 antibody (cHAb18) between the HN and F genes of the oncolytic Newcastle disease virus (NDV) by the reverse genetic system to form a recombinant NDV carrying CHAb18 (rNDV-18HL) maintained the same virulence as the wild NDV strain while additionally attained optimal active cHAb18 expression with increased *in vitro* and *in vivo* cytotoxic and anti-metastatic activities against HCC SMMC-7721 cell line (106).

Using cHAb18 as a combined or adjuvant to conventional chemoradiotherapies is another potential role by CD147. *In vitro* and *in vivo* studies on the combination of metuzumab with the nucleoside-derived chemotherapies, gemcitabine and cytarabine (Ara-C) have revealed promising results: *in vitro* and *in vivo* study under ^18^F-FDG PET/CT of combined gemcitabine/cHAb18 therapy in MIA PaCa-2 pancreatic cancer cell line showed an additively superior cytotoxic activity than either of the two therapies alone evidenced by relatively decreased Ki-67 expression (*p=0.008*) (121); while in NSCLC, combining metuzumab and gemcitabine enhanced gemcitabine-induced cytotoxicity, G1 cell cycle arrest, and upregulation of the nucleoside salvage pathway protein, deoxycytidine kinase (dCK) responsible for converting gemcitabine into active nucleotides gemcitabine diphosphate (dFdCDP) and triphosphate (dFdCTP)) (102). Cytosine arabinose (Ara-C, cytarabine) is commonly used to treat acute myeloid leukemia (AML) but with significant treatment failure like that mediated by Glioma-associate protein 1 (GLI1, a sonic hedgehog transcription factor) (122); Treatment of gastric cancer cell line, AGS, with Ara-C was shown to result into heightened invasiveness through extracellular signal-regulated kinase (ERK)-mediated upregulation of HAb18G downstream factors, MMP-2 and MMP-9, but inhibition of CD147 via siRNA halted the Ara-C-induced invasiveness and restored antiproliferative activity of Ara-C (80), an evidence of therapeutic potential by targeting CD147 to reverse Ara-C resistance in cancers. Additionally, chemo-radio-resistance common in pancreatic cancer stem cells was reversed by treatment with cHAb18 which was effected through blockage of CDs44-STAT3 pathway and reduction of transcription factors NANOG, OCT4, and SOX4, rendering the cells sensitive to gemcitabine-cHAb18 combined therapy (44).

## 5 Conclusion and future perspectives

CD147 is a molecule critically involved in the acquisition and propagation of hallmarks of cancer and cancer-enabling characteristics. It is therefore one of the key driver molecules in cancer development, microenvironment adaptation, progression, metastasis, and resistance to therapies witnessed in many human cancers.

Because CD147 promotes cancer progression and metastasis through multiple signaling pathways, a single block of CD147 would therefore translate into fissional phenomena of arresting multiple and alternate cancer signaling cascades; this ensures minimal treatment-evasion of cancer cells that would result from epigenetic modulations, mutations of a single or multiple pro- and anti-oncogenes, and supplementary molecules that interplay within the hallmarks of cancer. Since CD147 is differentially expressed in cancer cells than in normal tissue cells, targeted therapies as well as immunotherapies against CD147 are therefore less likely to attack normal tissue and organs hence avoiding severe adverse events, this being one of the significant challenges with current immunotherapies. In that light, further experimental and clinical studies have a promising potential of yielding efficacious and safe treatment against aggressive human cancers that remain challenging to date.

## Author contributions

FL, AN, AL, and YX developed conceptualization for the study. AN, HL, TR, XS, and JZ performed literature curation and data analysis. FL was responsible for funding acquisition and project administration. FL, AN, and HL oversaw resource distribution and supervision throughout the study. AN, AL, XZ, TR, and JZ performed information synthesis and validation. FL, AN, and XS managed visualization designs. The original draft was written by FL, AN, AL, YX, TR, and XZ. All authors contributed to the article and approved the submitted version.
